# Concomitant Gastric Leak, Portal Vein Thrombosis, and Liver Abscesses Following Sleeve Gastrectomy: A Report of a Rare Case

**DOI:** 10.7759/cureus.73813

**Published:** 2024-11-16

**Authors:** Karla Carolina Flores-Maciel, Nahomi Sharon Siordia-Cruz, Luis Osvaldo Suárez-Carreón

**Affiliations:** 1 General Surgery, American British Cowdray Medical Center, Mexico City, MEX; 2 General Surgery, Mexican Social Security Institute, Specialty Hospital, Western National Medical Center, Guadalajara, MEX; 3 Bariatric Surgery, Mexican Social Security Institute, Specialty Hospital, Western National Medical Center, Guadalajara, MEX

**Keywords:** bariatric surgery, complications, leak, liver abscesses, portal thrombosis

## Abstract

One of the most serious complications after sleeve gastrectomy (SG) is a postoperative leak. Early diagnosis and treatment are essential due to potential secondary complications, such as sepsis, septic shock, and death. Less commonly known and rare complications include portal thrombosis and liver abscesses, which have been reported in only a few cases. Here, we present a patient who developed a leak, portal thrombosis, and concomitant liver abscesses, requiring both surgical and medical management, ultimately leading to a favorable outcome. To our knowledge, this is the first case report of these three concomitant complications in a patient in Mexico.

## Introduction

Sleeve gastrectomy (SG) is the most widely performed bariatric procedure globally and is an effective treatment for obesity [1]. However, it is not without complications. These complications are classified as early (within 30 days) or late (after 30 days). The most frequent complication is bleeding, with an incidence of 1.16-4.94% [2]. One of the most serious complications is gastric leak, occurring in 1-3% of primary procedures and up to 10% in revision procedures [2]. Leaks can occur anywhere along the staple line, with 75-85% occurring proximally near the angle of His. This is likely due to increased intragastric pressure caused by altered peristalsis and ischemia [2,3]. The occurrence of leaks is associated with surgical technique modifications (e.g., suture reinforcement, distance from the pylorus, size of the gastric tube) and the presence of metabolic syndrome components, especially type 2 diabetes mellitus [2]. The clinical presentation can vary from asymptomatic (detected on contrast studies) to severe, with peritonitis, septic shock, multiple organ failure, and death [2]. Tachycardia is often the initial sign of a gastric leak. Chronic leaks are defined by a fistulous tract with continuous output after six months despite treatment. These leaks may drain into an intra-abdominal collection or adjacent structures. Treatment typically involves prolonged recovery and a complex multidisciplinary approach [2].

Another potentially fatal but infrequent complication is thrombosis or thrombophlebitis of the portal, splenic, or mesenteric veins. This can result in gastrointestinal ischemia and infarction. The pro-inflammatory and hypercoagulable states associated with obesity predispose patients to thromboembolic complications. This complication is reported in 0.3-1% of post-bariatric surgery patients, being more frequent after SG [4]. The portal vein is most commonly affected, followed by the superior mesenteric and splenic veins. Thrombosis has occluded 100% of the affected vessels in most cases, with 88% of cases occurring within the first month postoperatively. This may be due to the potential mechanisms proposed by Goiten et al. that contribute to the increased risk of portomedenteric vein thrombosis after SG, which include altered blood flow post-SG due to the division of the short gastric vessels, direct physical contact with the splenic vein while working on the omental bursa, and early postoperative discharge leading to dehydration [5]. Patients may present with vague symptoms, such as abdominal pain, nausea, vomiting, or leukocytosis, necessitating a high index of suspicion for diagnosis [4]. The current treatment is anticoagulation, initiated immediately upon diagnosis, as recanalization occurs in more than 80% of cases [6]. Treating predisposing conditions is also crucial to ensure vessel recanalization and prevent recurrence [4]. Serious consequences, such as portal hypertension and its sequelae (ascites (62%), esophageal varices (58%), gastroesophageal bleeding (47%)), cirrhosis, and intestinal infarction, can result from this complication [6].

A third complication after SG is liver abscess formation, which can lead to sepsis, empyema, and peritonitis, making early diagnosis critical. The two possible risk factors for this complication are bacterial migration secondary to gastric leak and bacterial migration from the portal venous system due to pylephlebitis of the portomesenteric veins [7]. First-line treatment involves percutaneous aspiration with empirical antibiotic therapy, later guided by culture results [7,8]. This case report presents a patient with all three complications occurring concomitantly, marking the first such case to be reported in Mexico, and emphasizes the importance of early detection of the complication that we believe was the trigger for the rest of them, which was the gastric leak.

## Case presentation

**Figure 1:**
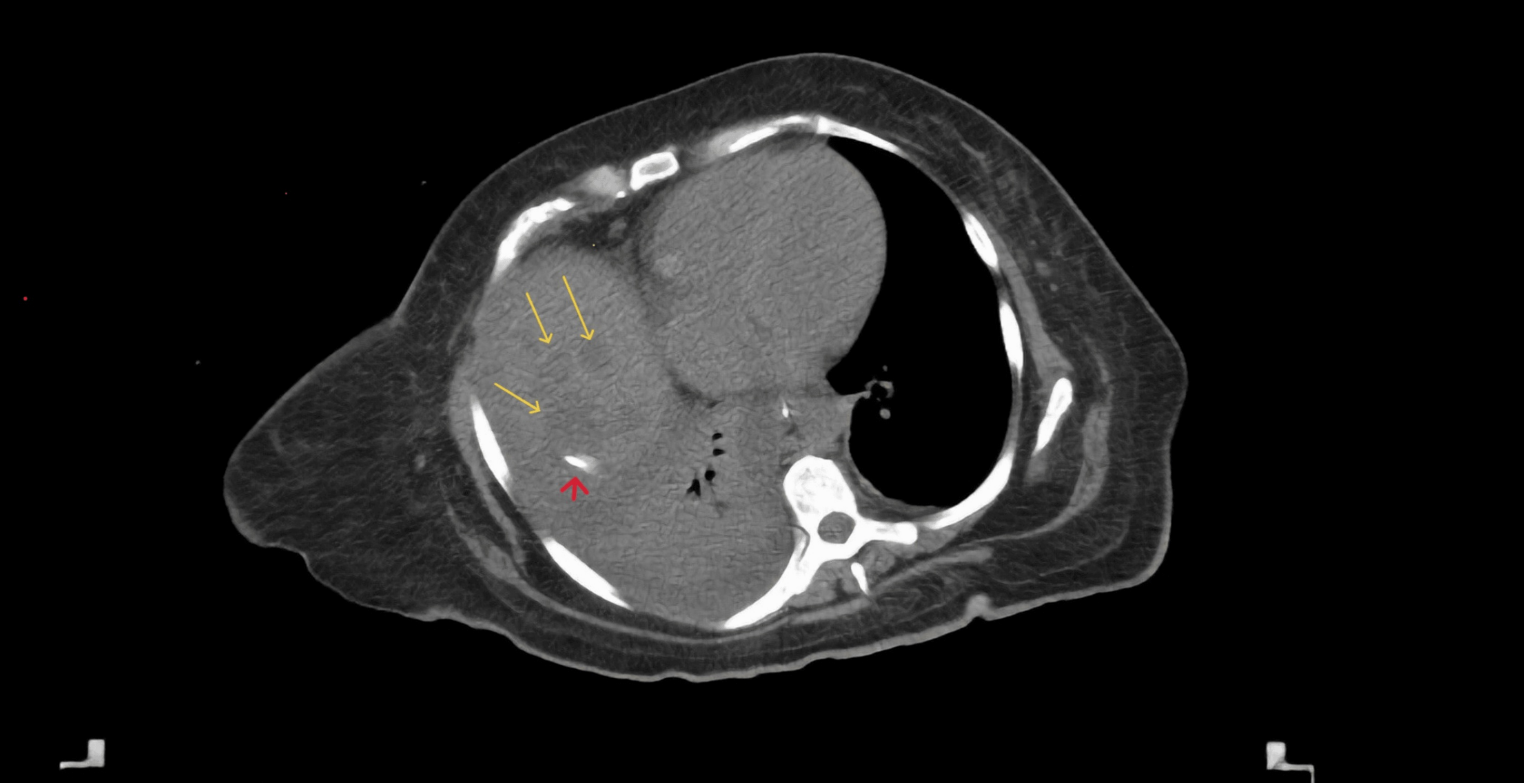
Abdominal CT scan CT scan taken upon admission, showing multiple hypodense lesions in segment VII suggestive of liver abscesses (yellow arrows) and the drain in the biggest abscess (red arrow).

**Figure 2:**
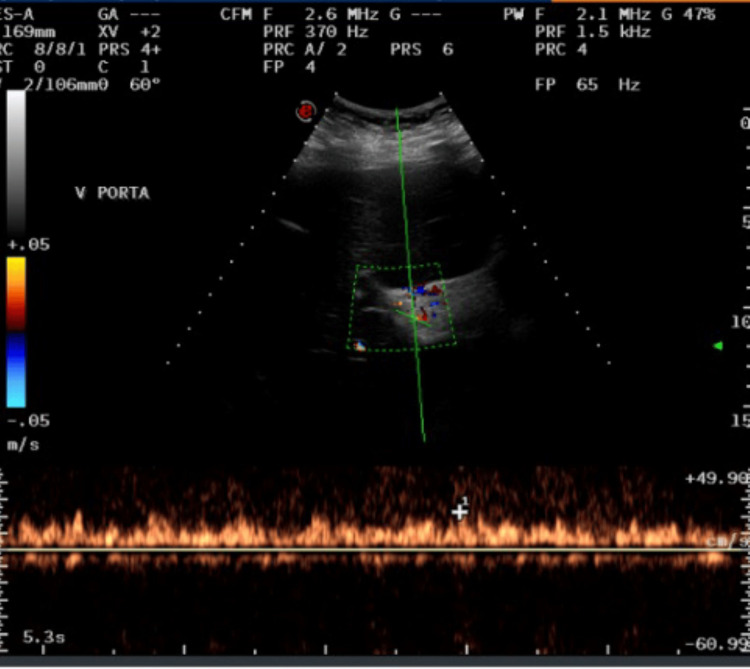
Doppler ultrasound of portal vein Doppler ultrasound demonstrating 100% thrombosis of the portal vein.

**Figure 3:**
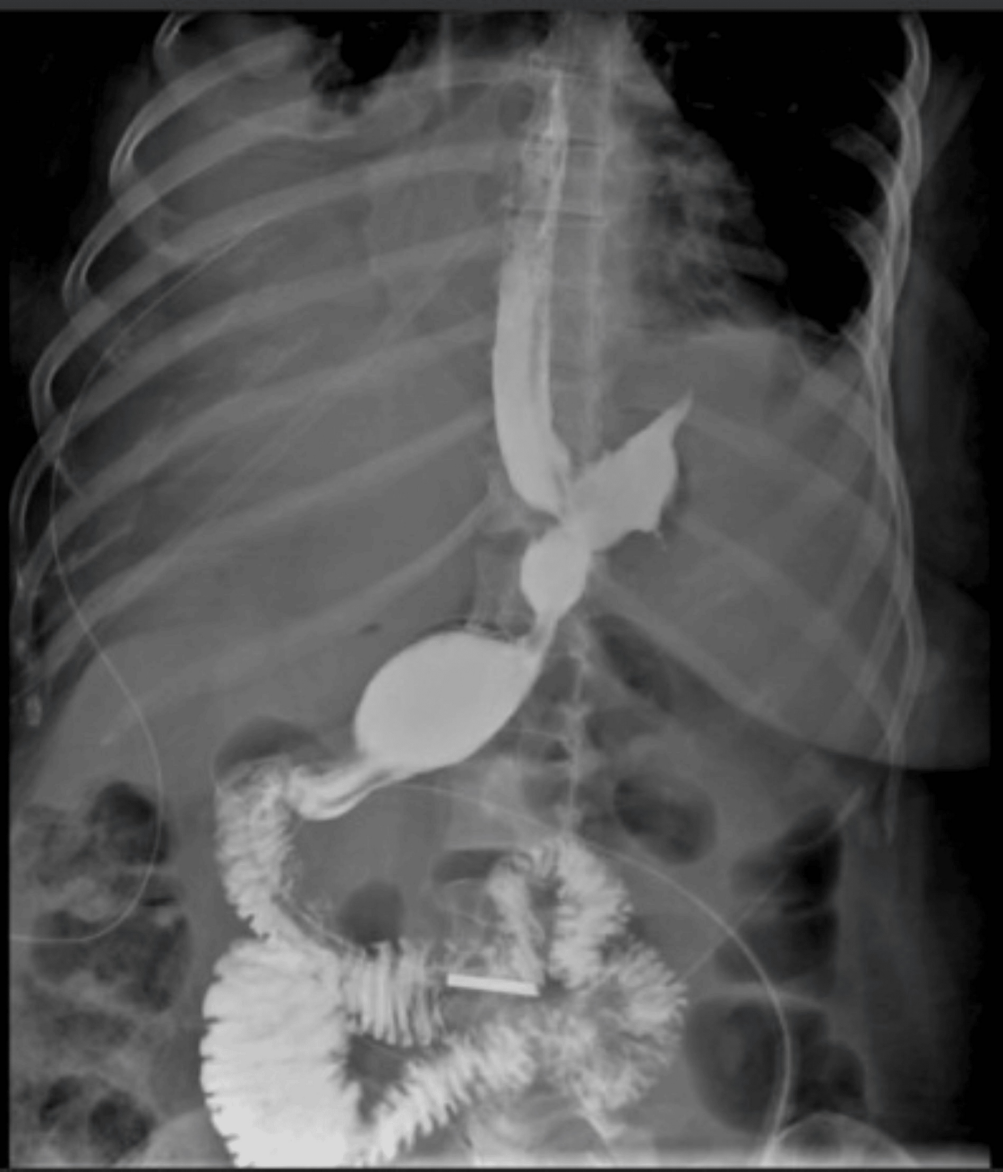
Esophagogastric series Esophagogastric series demonstrating absence of leaks.

**Figure 4:**
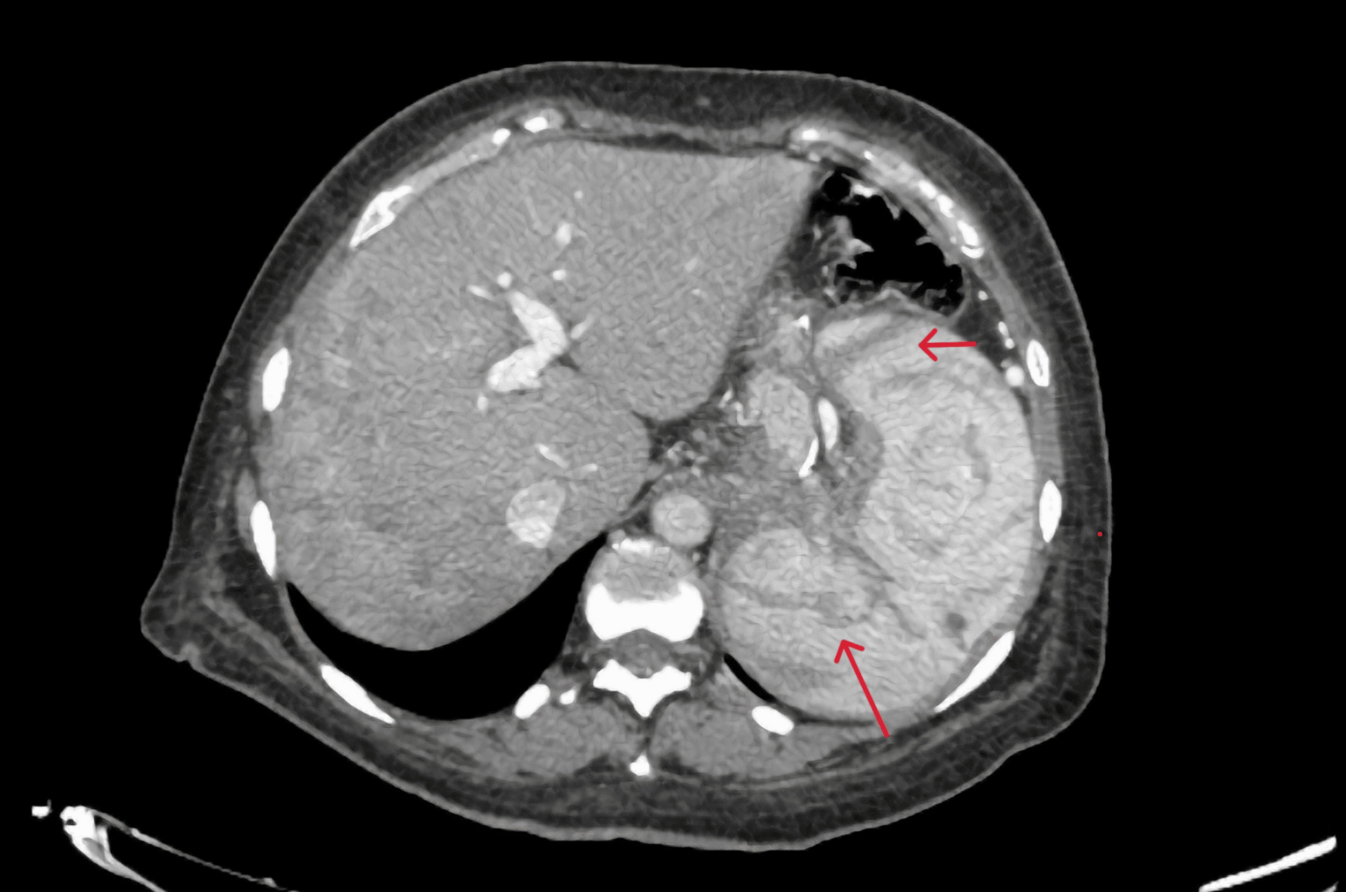
Follow-up abdominal CT scan CT scan with IV contrast showing heterogeneous enhancement in liver parenchyma, without abscesses. Multiple areas of splenic infarction (red arrows).

A 45-year-old female patient with a history of type 2 diabetes mellitus, asthma, grade 1 obesity, and smoking underwent SG five months before the admission and received a course of 7 days of antibiotic therapy after the surgery. She presented with fever, general malaise, and shock to the private hospital where she had her SG before, and a gastric leak was suspected. An exploratory laparoscopy was decided where the leak was confirmed, and hepatic, perigastric, and perisplenic abscesses were drained. After surgery, she was treated with broad-spectrum antibiotics and total parenteral nutrition with a good response. Twelve days later, she was referred to our center, where a CT scan revealed at least three hypodense lesions suggestive of liver abscesses in segment VII, the largest measuring 87 × 60 mm, with a drain in place (Figure 1). Doppler ultrasound showed complete portal thrombosis with collateral vessels (Figure 2). A gastroduodenal esophagus series with water-soluble contrast demonstrated no leaks (Figure 3). We initiated an enteral diet through a nasojejunal tube and broad-spectrum empirical antibiotics. The patient was evaluated by angiology for portal thrombosis but was not a candidate for endovascular procedures due to the chronicity of the thrombosis. She was managed with anticoagulation (low molecular weight heparin) and close monitoring. Blood cultures revealed Peptostreptococcusspecies, and targeted antibiotic therapy was administered for four weeks. Drains were removed after four weeks, and the patient was discharged with oral anticoagulation (rivaroxaban) for six months and oral antibiotics. At eight-month follow-up, she was asymptomatic and tolerating oral intake. The follow-up endoscopy reported integrity of the gastric sleeve without alterations. Additionally, a new abdominal CT scan with intravenous contrast reported no abscesses in the liver parenchyma but areas of splenic infarction (Figure 4). The latest laboratory tests showed anemia (hemoglobin 11.2 g/dL), normocytic and normochromic, hematocrit 34.2%, leukocytes 4.39 × 10³/µL, and platelets 99 × 10³/µL. Coagulation times are slightly prolonged (prothrombin time (PT) 13/10.3 seconds, international normalized ratio (INR) 1.26, activated partial thromboplastin time (APTT) 33.2/29.6 seconds). Bilirubin levels were normal (total bilirubin (TB) 0.68 ng/dL, direct bilirubin (DB) 0.19 ng/dL, indirect bilirubin (IB) 0.49 ng/dL), as were transaminase levels (alanine aminotransferase (ALT) 53 U/L, aspartate aminotransferase (AST) 23 U/L, gamma-glutamyl transferase (GGT) 73 U/L), and albumin was 3.4 g/dL. The plan is to continue monitoring for complications associated with portal thrombosis, such as portal hypertension, variceal bleeding, and cirrhosis.

## Discussion

Complications following SG occur in up to 3% of cases, with gastric leak being the most serious due to its high mortality rate. Leaks can result from mechanical causes (e.g., poor stapling, tissue injury) or ischemic causes [7]. In our case, no evidence of a gastric leak was found in the studies performed, suggesting that it was resolved with surgical drainage. However, late presentation of a leak is rare, and clinical suspicion, as well as close monitoring of patients, is of utmost importance. Early treatment can prevent life-threatening complications such as those presented in our case.

Liver abscess is an infrequent complication with various etiologies, such as an enteric fistula, bacterial dissemination from infected material in the gastrosplenic area, and bacterial migration through the portal venous system secondary to pylephlebitis of the portomesenteric veins. Although we cannot know which complication came first in our case, all these potential causes of liver abscess were present [9]. Clinical manifestations typically include right upper quadrant abdominal pain, fever, and general malaise. Approximately 25% of patients exhibit jaundice and weight loss. CT with IV contrast is the diagnostic modality of choice, with a sensitivity of 95-97% [10]. Abscesses appear as well-defined lesions with peripheral enhancement and internal septa. Blood cultures should be obtained before initiating antibiotics, and early antibiotic treatment is critical to reduce the risk of septicemia and systemic complications. Small abscesses (<3-4 cm) can be managed with antibiotics alone, with a success rate close to 100%. Larger abscesses or those not responding to antibiotics within five to six days may require percutaneous or surgical drainage. Surgical intervention is indicated for abscesses >5 cm, multiple or multiloculated abscesses, or complications such as peritonitis or abscess rupture [10]. The treatment of the abscesses in our patient was combined: surgery to drain the largest abscess and, for the smaller ones, intravenous antibiotic therapy for six weeks with a favorable resolution.

Patients undergoing bariatric surgery are at high risk of thrombosis. Portomesenteric thrombosis, a serious complication increasingly reported after bariatric surgery, was first described following splenectomy. This condition can result from direct trauma to the portomesenteric circulation, blood flow stasis due to ligation of gastrointestinal vessels, or an inflammatory response secondary to laparoscopy. Increased pneumoperitoneum and the reverse Trendelenburg position in severely obese patients may further elevate the risk. Portomesenteric thrombosis can occur between one and 2,569 days postoperatively, with most cases occurring within the first month [4]. In our case, thrombosis occurred 150 days after SG. Portomesenteric thrombosis is classified as primary (idiopathic) or secondary (due to an identifiable cause). Early detection is crucial, as symptoms are often vague, and physical examination findings are non-specific. Delayed treatment can lead to thrombus organization with secondary portal cavernomatosis, potentially causing complications such as variceal bleeding [11]. Doppler ultrasound is the diagnostic test of choice. Acute thrombosis appears as a hypoechoic or isoechoic area within the vessel, while chronic thrombosis appears hyperechoic, with Doppler showing partial or complete filling defects. Other diagnostic tools include contrast ultrasound, CT with IV contrast (preferred for chronic thrombosis), and MRI [12].

Treatment should begin immediately upon diagnosis. The goal of anticoagulation in acute thrombosis is to limit thrombus extension and facilitate vessel recanalization, thereby preventing complications like portal hypertension and intestinal infarction [12]. In stable patients without evidence of intestinal ischemia, anticoagulation may suffice, with surgery reserved for those who develop acute abdominal signs or symptoms. Anticoagulation can achieve partial recanalization in 63-97% of patients and complete recanalization in 34-45%. Initial treatment typically involves low molecular weight heparin or unfractionated heparin, transitioning to oral anticoagulants once the patient is stable and has no upcoming invasive procedures. Treatment continues for three to six months or indefinitely in patients with uncorrectable procoagulant conditions or thrombosis extending to mesenteric veins, as in our patient [12,13]. Other options include thrombolysis with streptokinase for patients unresponsive to anticoagulation, though the evidence is limited [12].

In this case, we want to emphasize the importance of early detection of complications. We believe that the sequence of events was first a gastric leak with the formation of an intra-abdominal collection that led to portal vein thrombosis and that later, due to septic emboli, liver abscesses formed. Although we cannot know with precision, another option is that first, there was a leak, then there were abscesses due to continuity, and finally, there was thrombosis. However, if the initial complication had been detected, the following complications that could have ended the patient's life could have been avoided. In this case, we also want to share the treatment and the good evolution of the patient so that it can serve as a reference in case of witnessing these complications together.

## Conclusions

In conclusion, the occurrence of gastric leak, portal thrombosis, and liver abscesses after SG is rare, and even rarer is their concomitant presentation due to a delayed leak diagnosis. Each complication requires specific treatment, which, in this patient, includes surgical drainage, prolonged antibiotic therapy, and anticoagulation. This case underscores the importance of early detection and treatment of gastric leaks following SG to prevent a cascade of preventable complications, which we already mentioned in our case and can threaten a patient's life. We suggest that further research is needed with prospective studies of patients presenting with late leakage to look for other risk factors for these complications, as well as long-term studies to recommend the most appropriate treatment, surveillance, and prognosis.
